# Development of glass micro-electrodes for local electric field, electrical conductivity, and pH measurements

**DOI:** 10.1038/s41598-020-60713-z

**Published:** 2020-03-05

**Authors:** Kentaro Doi, Naoki Asano, Satoyuki Kawano

**Affiliations:** Department of Mechanical Science and Bioengineering, Graduate School of Engineering Science, Osaka University, Toyonaka, Osaka, 560-8531 Japan

**Keywords:** Chemical physics, Chemical engineering

## Abstract

In micro- and nanofluidic devices, liquid flows are often influenced by ionic currents generated by electric fields in narrow channels, which is an electrokinetic phenomenon. Various technologies have been developed that are analogous to semiconductor devices, such as diodes and field effect transistors. On the other hand, measurement techniques for local electric fields in such narrow channels have not yet been established. In the present study, electric fields in liquids are locally measured using glass micro-electrodes with 1-μm diameter tips, which are constructed by pulling a glass tube. By scanning a liquid poured into a channel by glass micro-electrodes, the potential difference in a liquid can be determined with a spatial resolution of the size of the glass tip. As a result, the electrical conductivity of sample solutions can be quantitatively evaluated. Furthermore, combining two glass capillaries filled with buffer solutions of different concentrations, an ionic diode that rectifies the proton conduction direction is constructed, and the possibility of pH measurement is also demonstrated. Under constant-current conditions, pH values ranging from 1.68 to 9.18 can be determined more quickly and stably than with conventional methods that depend on the proton selectivity of glass electrodes under equilibrium conditions.

## Introduction

In recent decades, ion transport phenomena that occur in micro- and nanofluidic channels have often been applied to micro-electromechanical systems (MEMS), where the surface area becomes more important than the volume^1–5^. Surface effects sometimes contribute to effective heat and mass transfer in liquids. As described in the literature, electromagnetic fields applied in micro- and nanochannels also induce peculiar electrokinetic transport phenomena^6–9^. Electrically charged particles are driven by the Coulomb force and viscous drag of a liquid flow that is caused by electroosmosis^3,8^. Using such a specific situation, particles and molecules translocate in micro- and nanochannels toward a test section in which single particle characteristics are sensed^1,2,5,10,11^. This kind of technology is expected to expand in various research fields, such as physics, chemistry, biology, and medicine^2,4,8^.

In a previous study, we investigated the transport of deoxyribonucleic acid (DNA) in nanochannels that have negative charges in electrolyte solutions^12–14^. By applying an potential difference, DNA molecules were found to be transported by electrophoresis in an electroosmotic flow. Although it was clear that the behavior of electrically charged molecules was actually influenced by the externally applied field, quantitative evaluation of the local electric field in the channel was difficult. Some numerical studies predicted an potential difference in electrolyte solutions as a function of ion concentration^14–17^. By solving the Nernst-Planck and Poisson equations, the relationship between the electrical potential and the concentration was clarified^18,19^. In particular, an electrode surface is strongly screened by electrolyte ions that form an electric double layer (EDL), and thus the applied electrical potential usually decreases near the surface. The thickness of the EDL is usually too small to measure experimentally. This is the reason why experimental methods to clarify such local fields in liquids have not yet been fully established. In research on sensory organs, understanding the relationship between electric fields and ion concentrations is important because the response of the sensors to external stimuli propagates in lymphatic liquids via nerve cells, where ion concentration differences between perilymph and endolymph liquids generate electrical signals^20,21^. However, the detailed behavior of spatially distributed ions has not yet been clarified.

In the present study, we carry out experimental measurements of the local electric field in electrolyte solutions with a resolution of 1 μm, based on the diameter of the glass capillary. By translocating a micro-glass electrode in electrolyte solutions, the spatial distribution of the electrostatic potential in ionic current conditions is quantitatively clarified. The electric field at the connection of channels with different cross-sectional areas changes according to the concentration of electric lines of force. Although this type of phenomenon has already been suggested by some numerical analyses^22–24^, it is novel to elucidate the electric field in actual experimental systems. In a narrow channel in which an almost uniform electric field is formed, the current-voltage (*I*-*V*) characteristics are linearly analogous to Ohm’s law. Taking the gradient of the *I*-*V* characteristics, the conductance of electrolyte solutions is also quantitatively evaluated. It is important to design a suitable channel and to confirm the existence of a uniform electric field. The present method may be used to evaluate the local field in the entire system. Furthermore, we propose the development of the present method using double-barreled glass micro-electrodes for the ionic diode and pH sensor.

**Figure 1:**
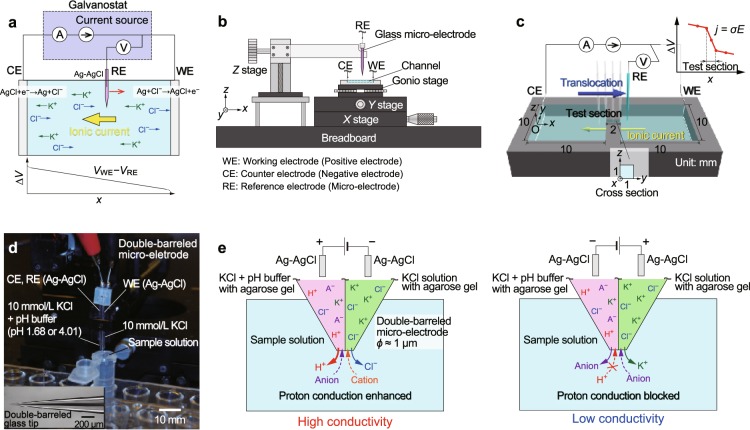
Schematic diagram of local electrical potential measurement by translocating the reference electrode of a galvanostat (**a**). Experimental apparatus in which a sample solution placed on an *XY* stage is translocated along the *x*-axis, and the potential difference is monitored using a glass micro-electrode (**b**). Dimensions of a channel that has a narrow test section to measure uniform electric fields applied to electrical conductivity measurement (**c**). Photograph of the double-barreled glass micro-electrode used as a pH sensor based on the principle of the glass micro-electrode (**d**). Schematic explanation of a pH sensor using a double-barreled glass micro-electrode by which asymmetric *I*-*V* characteristics are obtained depending on the direction of proton conduction due to the proton concentration difference between the inner and outer solutions (**e**).

Ionic diodes and pH sensors have a long history of development. Hinke^25^ performed pioneering work in measuring the activity of sodium and potassium in the squid axon using glass micro-electrodes. He demonstrated that glass micro-electrodes had cation selectivity. Thomas *et al*.^26,27^ developed a glass micro-electrode that enabled measurement of the pH of liquids due to the proton selectivity of glass capillary tips. This is the basic principle of the pH sensor that is widely used today. In other research fields, ion-sensitive field-effect transistors (ISFETs) have been developed, e.g., ionic diodes and ionic transistors^9,28–34^. ISFETs are based on a pn junction formed in liquids due to surface effects in narrow channels, where the surface charge of the channel walls determines the dominant charge carriers in liquids. The connection of narrow channels that have positively and negatively charged surfaces produces a diode that can be opened or closed under an applied electrical potential^7,28,29^. Cheng *et al*.^35,36^ successfully implemented a microscale pH sensor in microfluidic devices using ion-exchange materials. In the present study, we also demonstrate diode characteristics and pH measurements using a double-barreled glass micro-electrode based on the same principle of conductivity measurement. As shown in Fig. 1, the details of which will be explained later, the proposed method may not depend on the surface effects of materials, but rather on the structure of the electrodes that separate the inner and outer solutions, resulting in ion selectivity due to the enhancement and blockage of proton conduction at the interface between the inner and outer solutions. The present method is simple and easy to use to measure various properties of electrolyte solutions, and is expected to be applied in various research fields, e.g., electrokinetics, physiology, biology, and physical chemistry.

## Results and Discussion

### Local electric field measurement using the glass micro-electrode

**Figure 2:**
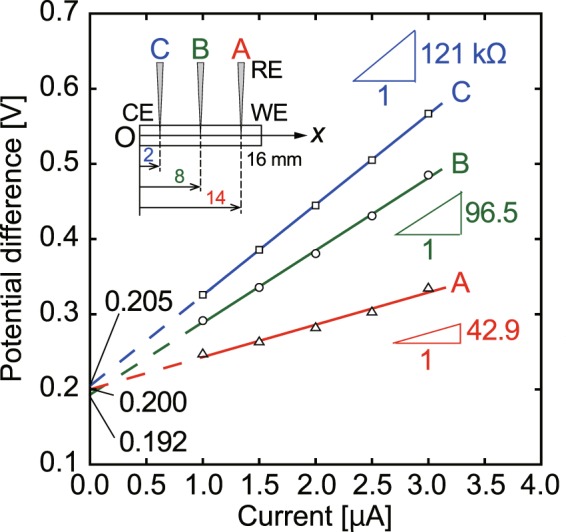
Experimental results for potential difference as a function of ionic current measured in a channel 16 mm in length, 2 mm in width, and 3 mm in depth, in which the counter electrode (CE) and working electrode (WE) are at *x* = 0 and 16 mm, respectively, and the glass micro-electrode (RE) is translocated along the *x*-axis. Measurement points are located at *x* = 2 mm (A), 8 mm (B), and 14 mm (C).

First, we introduce the preliminary results of electrical potential measurement using the glass micro-electrode in a straight channel 16 mm in length, 2 mm in width, and 3 mm in depth. There were three measurement points fixed at the center in the 16 mm long channel and at ±6 mm from the center. A constant ionic current was maintained between the working electrode (WE) and counter electrode (CE) at both ends of the channel filled with a 1.0 × 10^−3^ mol/L KCl solution. As shown in Fig. 2, we obtained linear *I*-*V* characteristics at each point, applying a constant current ranging from 1.0 to 3.0 μA, which resulted in a conductivity of 2.76 × 10^−2^ S/m at the center of the channel. The electrical conductivity of the standard solution of 1.0 × 10^−3^ mol/L KCl is 1.4695 × 10^−2^ S/m^37,38^, so the present result evaluated at the center of the channel extremely overestimated the conductivity. This large difference may be caused by the assumption of a uniform electric field, because in the actual system, the electric field in a liquid is influenced by screening of the electrode surface and a non-uniform ion distribution. Therefore, we need to determine the spatial distribution of the electrical potential in the liquid and use a test channel that has a narrow test section, as shown in Fig. 1c. The test section has a 1 mm × 1 mm cross section and a length of 2 mm. Translocating the glass micro-electrode in this test section, the potential difference was locally measured. Some typical results for the potential difference as a function of the micro-electrode position are compared with the numerical analysis later. The concentration of the KCl solution was varied as 5.0 × 10^−4^, 1.0 × 10^−3^, 5.0 × 10^−3^, and 1.0 × 10^−2^ mol/L. The electrical potential is seen to exhibit a steep constant slope in the 2-mm test section, which indicates the presence of a uniform electric field in the solution. Regardless of the solution concentration, a strong electric field is present in the test section under ionic current conditions. The experimental results closely agree with the numerical results. The electrical conductivity was evaluated based on the salt concentration and the electric field in the test section, and the results are summarized in Table 1. The present results of 7.93 × 10^−3^, 1.67 × 10^−2^, 6.92 × 10^−2^, and 1.46 × 10^−1^ S/m, which were respectively obtained for the 5.0 × 10^−4^, 1.0 × 10^−3^, 5.0 × 10^−3^, and 1.0 × 10^−2^ mol/L KCl solutions, well reproduced the electrical conductivity of KCl standard solutions, and clearly exhibited linearity. The high resistance in the test section of the channel is thought to cause the large potential drop and the uniform electric field. Consequently, the electrical conductivity can be measured analogous to Ohm’s law.

**Table 1:** Conductivity of various KCl solutions determined by local electric field measurements.

*c*_0_ [mol/L]	Conductivity [S/m]		Difference [%]
	Present	Reference^37,38^	
5.0 × 10^−4^	7.93 × 10^−3^	7.3905 × 10^−3^	7.3
1.0 × 10^−3^	1.67 × 10^−2^	1.4695 × 10^−2^	13.6
5.0 × 10^−3^	6.92 × 10^−2^	7.1775 × 10^−2^	3.6
1.0 × 10^−2^	1.46 × 10^−1^	1.4127 × 10^−1^	3.5

**Figure 3:**
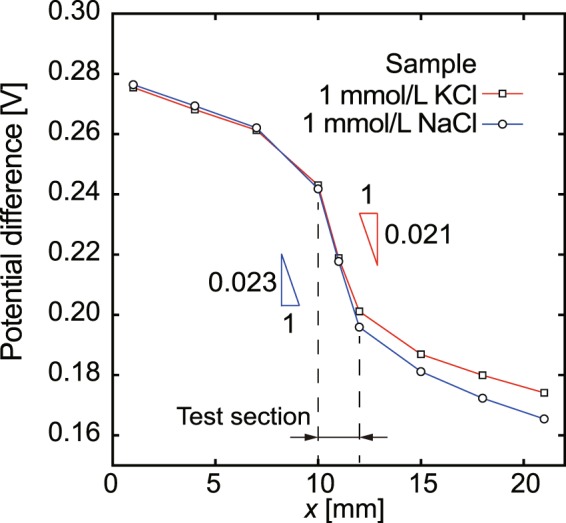
Experimentally obtained electrical potential curves for 1.0 × 10^−3^ mol/L KCl and NaCl sample solutions using a micro-electrode with a 1.0 × 10^−1^ mol/L KCl inner solution.

**Figure 4:**
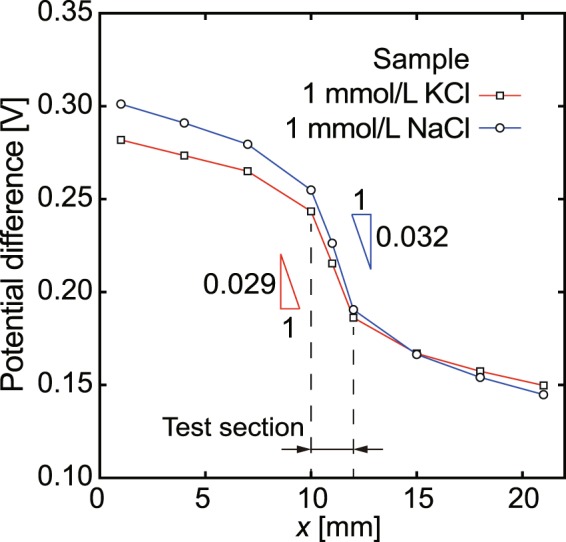
Experimentally obtained electrical potential curves for 1.0 × 10^−3^ mol/L KCl and NaCl sample solutions using a micro-electrode with a 1.0 × 10^−1^ mol/L NaCl inner solution.

Next, we investigated the possibility of characterization of different types of electrolyte solutions using the glass micro-electrode. A sample of 1.0 × 10^−3^ mol/L NaCl solution was measured by the micro-glass electrode in 1.0 × 10^−1^ mol/L KCl. The obtained electrical potential curves are shown in Fig. 3. The electrical potential clearly dropped in the test section in the same way as in the case of the KCl samples. Furthermore, using a 1.0 × 10^−1^ mol/L NaCl solution for the inner solution of the glass micro-electrode, KCl and NaCl sample solutions of 1.0 × 10^−3^ mol/L were measured. As shown in Fig. 4, the electrical conductivity was measured in the uniform electric field in the test section. The measurement results are summarized in Table 2. Using the micro-electrode with a 1.0 × 10^−1^ mol/L KCl inner solution, the electrical conductivity was (1.40 ± 0.05) × 10^−2^ S/m for 1.0 × 10^−3^ mol/L KCl and (1.30 ± 0.04) × 10^−2^ S/m for 1.0 × 10^−3^ mol/L NaCl. The differences compared with the standard solutions were 4.7% and 5.1%, respectively. In addition, the electrical conductivity of a 1.0 × 10^−2^ mol/L NaCl solution was evaluated to be (1.31 ± 0.05) × 10^−1^ S/m, which represents a 10.5% difference to that for the standard solution. Although the linearity was clear, the difference increased with increasing concentration of NaCl. The 1.0 × 10^−1^ mol/L KCl solution used for the glass micro-electrode can analyze NaCl samples as effectively as KCl samples, and the difference in electrical conductivity between the KCl and NaCl samples can be distinguished. Using a 1.0 × 10^−1^ mol/L NaCl solution for the inner solution of the micro-electrode, the electrical conductivity of NaCl and KCl solutions were also quantitatively evaluated as shown in Table 2. The differences from the standard values are 3.5%, 3.4%, and 8.3% for 1.0 × 10^−3^ mol/L NaCl, 1.0 × 10^−3^ mol/L KCl, and 1.0 × 10^−2^ mol/L KCl, respectively. This result indicates that the NaCl solution can also be used with the micro-electrode, and shows similar results to the KCl inner solution. Further optimization of the geometry of the channel in order to concentrate the electric field more strongly in the test section may be possible. This is a subject for future study. Consequently, the glass micro-electrode that has a 1-μm diameter tip can be used to measure local electric fields under constant-current conditions. Due to the concentration of the electric lines of force in a narrow channel, an almost uniform electric field is formed in the test section, which is suitable for the measurement of the electrical conductivity of electrolyte solutions. Using this method, it is expected that ion concentration fields as well as electric potentials at specific environments, such as interfaces at different size channel connections and near surfaces of ion-exchange membranes where enrichment and depletion occur under ionic current conditions^39–41^, are clarified. We will tackle such a challenging topic in the near future.

**Table 2:** Experimental results for electrical conductivity for various concentrations of KCl and NaCl solutions measured using 1.0 × 10^−1^ mol/L KCl and NaCl inner solutions in the glass micro-electrode.

*c*_0_ [mol/L]	Conductivity		Difference [%]
	Present	Reference^37,38,51^	
**1.0 × 10**^**−1**^ **mol/L KCl for micro-electrode**			
1.0 × 10^−3^ (KCl)	(1.40 ± 0.05) × 10^−2^	1.4695 × 10^−2^	4.7
1.0 × 10^−3^ (NaCl)	(1.30 ± 0.04) × 10^−2^	1.2374 × 10^−2^	5.1
1.0 × 10^−2^ (NaCl)	(1.31 ± 0.05) × 10^−1^	1.1853 × 10^−1^	10.5
**1.0 × 10**^**−1**^ **mol/L NaCl for micro-electrode**			
1.0 × 10^−3^ (NaCl)	(1.28 ± 0.05) × 10^−2^	1.2374 × 10^−2^	3.5
1.0 × 10^−3^ (KCl)	(1.42 ± 0.05) × 10^−2^	1.4695 × 10^−2^	3.4
1.0 × 10^−2^ (KCl)	(1.53 ± 0.06) × 10^−1^	1.4127 × 10^−1^	8.3

### pH measurement using the double-barreled glass micro-electrode

**Figure 5:**
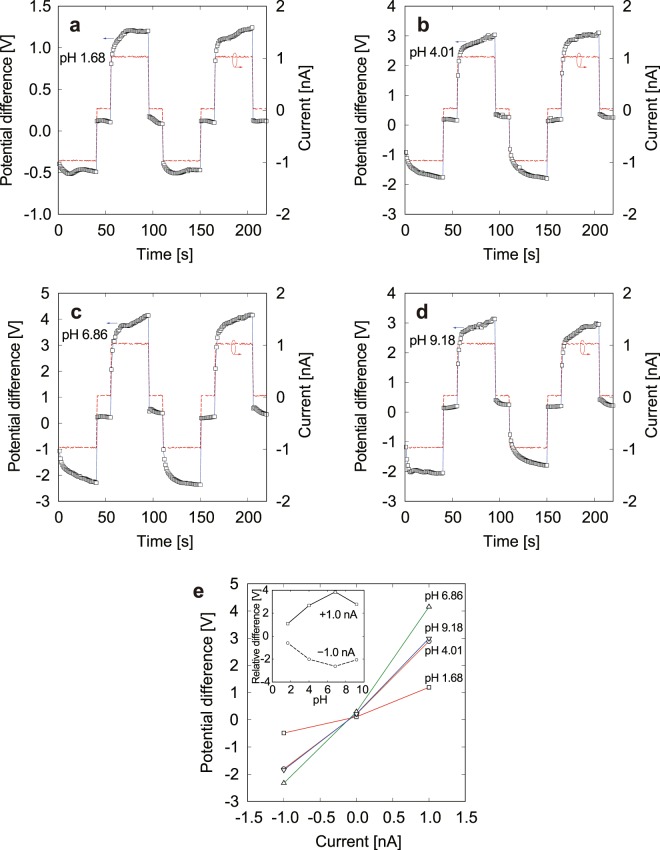
Response of potential difference (open squares) to ionic current changes (dashed lines) for samples with pH 1.68 (**a**), 4.01 (**b**), 6.86 (**c**), and 9.18 (**d**). The mixed solution in one side of the double-barreled glass micro-electrodes consists of 1.0 × 10^−2^ mol/K KCl and pH 4.01 buffer solutions and that in the other side is a 1.0 × 10^−2^ mol/L KCl solution. *I*-*V* characteristics associated with **a**–**d** (**e**). The potential differences at ± 1.0 nA relative to 0 nA as a function of pH are also shown in the inset.

**Figure 6:**
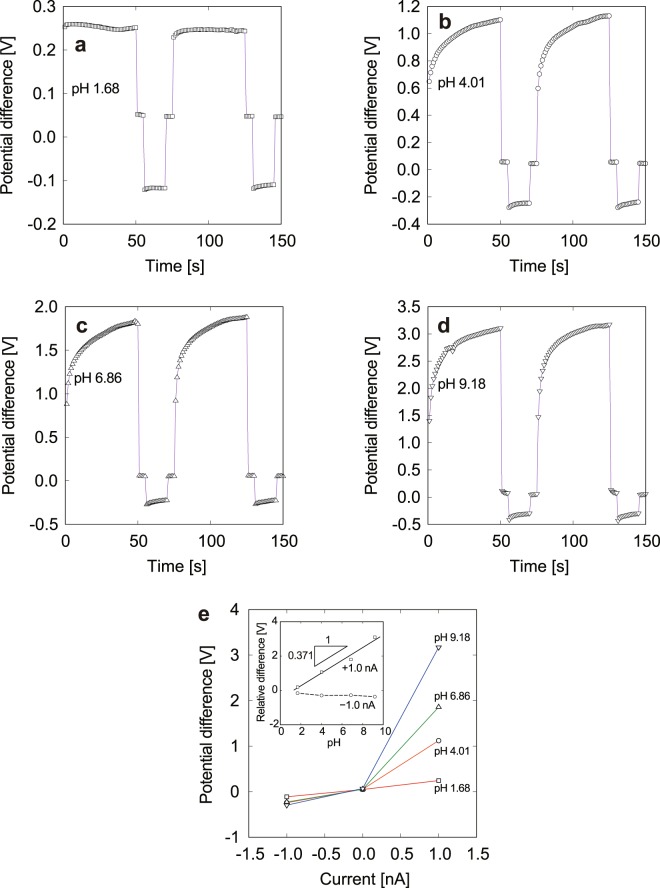
Responses of potential difference to ionic current changes for samples with pH 1.68 (**a**), 4.01 (**b**), 6.86 (**c**), and 9.18 (**d**). *I*-*V* characteristics measured using the double-barreled glass micro-electrode in which a mixture of 1.0 × 10^−2^ mol/L KCl and pH-1.68 buffer solutions are at one side and 1.0 × 10^−2^ mol/L KCl at the other side (**e**). Potential differences at ± 1.0 nA relative to 0 nA as a function of pH are also shown in the inset.

Based on the above quantitative evaluation of the electrical conductivity of KCl and NaCl solutions by measuring the local electrical potential, measurements of the pH were also conducted using the double-barreled glass micro-electrode. When the proton selectivity of the glass capillary tip is high, the electrical conductivity will depend mainly on the direction of proton conduction and diffusion caused by the concentration difference between the inner and outer solutions. As described in the methodology section, a constant ionic current was generated between a mixture of 1.0 × 10^−2^ mol/L KCl and pH 4.01 buffer solutions in one side of the double-barreled glass micro-electrode and a 1.0 × 10^−2^ mol/L KCl solution in the other side. Setting the double-barreled electrodes in a sample solution, a constant ionic current was maintained between the electrodes, and the time response of the potential difference was measured. Figure 5 shows typical responses obtained from sample solutions with pH values in the range from 1.68 to 9.18. As shown in Fig. 5, the response of the potential difference to the positive and negative currents was measured using sample solutions with pH 1.68 (**a**), 4.01 (**b**), 6.86 (**c**), and 9.18 (**d**). Pulsed currents of ±1.0 nA were applied for 40 s with an interval of 15 s. The potential difference shows a transient response immediately after the direction of the ionic current changes, and the potential converges to a constant value that depends on the pH. This is caused by the difference in proton conduction across the interface for solutions with different pH. The resistivity increases when protons are transported from the sample solution to the fixed-pH inner solution because the inner solution suppresses incoming protons. The converged potential difference as a function of ionic current is shown in Fig. 5e. In the *I*-*V* characteristics, the electrical conductance shows asymmetry in the direction of the ionic current. The difference in the *I*-*V* characteristics is caused by the electrical conductivity of the solutions. This result means that the sample solution has higher conductivity for a negative ionic current than a positive current. For the case of a sample solution with pH 6.86, the potential difference was −0.41 V for an ionic current of −1.0 nA and 3.84 V for an ionic current of 1.0 nA. When a negative potential relative to the reference electrode is applied to the working electrode in a 1.0 × 10^−2^-mol/L KCl solution in a mixed solution with pH 4.01, cations tend to be attracted to the working electrode, and protons that are highly concentrated in the mixture are conducted through the capillary to the sample solution. This direction is preferable for protons because the concentration difference between the inner and outer solutions is reduced. On the other hand, a reverse current suppresses diffusion of protons in the capillary of the low-pH mixed solution. This is why the electrical conductivity depends on the direction of the ionic current, as depicted schematically in Fig. 1e. Protons have an especially high conductivity compared to the other species^42^, and the *I*-*V* characteristics are governed by proton conduction in low-pH solutions. The pH difference between the solutions is suspected to be reflected in the electrical conductivity measured by the double-barreled glass micro-electrodes. Figure 5e shows the *I*-*V* characteristics for samples with various pH values. Although there is not so clear difference for the negative-current side, the electrical conductivity strongly depends on the pH value when the ionic current becomes positive. The potential difference for 1.0 nA increases for a pH of less than 7, and has a maximum value of 3.84 V at pH 6.89. On the other hand, the potential difference decreases for a pH of above 7. For a buffer with a pH of 9.18, the potential difference is 3.37 V, which is lower than that for the pH-6.86 buffer. It is suspected that increasing the pH above 7 causes an increase in the concentration of OH^−^ ions that also have high conductivity^43^. This might be the reason why the electrical conductivity of the buffer with pH 9.18 is higher than that for the buffer with pH 6.86, which had the lowest conductivity among the present solutions. In order to improve the order of pH values from strong acid to strong alkaline solutions in series, the double-barreled electrodes need to correctly measure the potential difference focusing only on proton conduction. Replacing the buffer with pH 4.01 in the mixed solution by an oxalate buffer with pH 1.68, the *I*-*V* characteristics for the samples were again measured using a procedure similar to that in the former experiment. The responses of potential difference to an electric current of ±1.0 nA are shown in Fig. 6a through 6d. In this case, a current of +1.0 nA was maintained for 50 s and a current of −1.0 nA was maintained for 15 s with an interval of 5 s. The potential difference is seen to clearly increase with increasing pH. As shown in Fig. 6e, the electrical conductance shows clear asymmetry in the direction of the ionic current, as in a diode, and ordered as a function of the pH in the positive-current domain. The relative difference in the electrical potential as a function of pH exhibited linearity, and the resolution was 371 mV/pH. This result improved the Nernst limit of 59 mV/pH that was measured at an equilibrium condition^44,45^. It is indicated that an increase in the proton concentration in the inner mixed solution decreased the electrical conductance of alkaline samples and that the potential difference produced by the ionic current was mainly governed by proton transport, suppressing the conduction of OH^−^ ions that become dominant at a pH of above 7. As described above, the present measurement method depending on the *I*-*V* characteristics via the double-barreled glass micro-electrodes showed stable responses under bias-current conditions. This is advantageous for reducing the time required to obtain constant pH values. Recently, micro- and nanoscale pH sensors have attracted much attention because of interests in the measurements of narrow spaces with higher accuracy, focusing on the application of biosensors^45,46^, flexible wearable devices^47,48^, and low cost and high throughput^44,49^. It is expected that the present results contribute to the development of such novel pH measurement techniques.

## Conclusions

In the present study, we developed a glass micro-electrode that was constructed by pulling a glass tube to a diameter of 1 μm. Using this capillary with Ag-AgCl wire electrodes, the potential difference in electrolyte solutions was locally measured under galvanostatic conditions. By concentrating the electric lines of force in a narrow test section embedded between wider reservoirs, a uniform electric field was obtained in the test section and the electrical conductivity of electrolyte solutions was quantitatively evaluated. The glass micro-electrode prepared with a 1.0 × 10^−2^-mol/L KCl solution could distinguish the difference in electrolyte species, such as KCl and NaCl, as well as the electrical conductivity. Further development of the double-barreled micro-electrodes allowed pH measurement based on the difference in proton conductivity between the fixed-pH mixed solutions and samples. The present double-barreled electrodes using an electrolyte solution with pH 1.68 succeeded in sensing the difference in proton conduction characteristics ranging from pH 1.68 to 9.18 with a resolution of 371 mV/pH.

The present principles may be applicable to micro- and nanochannel devices to measure local concentration profiles by designing miniaturized electrodes embedded in channels. Further applications to physiological measurement systems are also expected.

## Materials and Methods

### Glass micro-electrode for local electric field measurement

As shown in Fig. 1a, an electrolyte solution was poured into a channel with an electrode at each end to produce a constant ionic current. This channel was embedded in an acrylic substrate and placed on an *XY* stage to shift along the *x*-axis, as shown in Fig. 1b. The reservoirs at both ends of the test section had dimensions of 10 mm in width, 10 mm in length, and 1 mm in depth, as shown in Fig. 1c. The test section was 1 mm wide, 2 mm long, and 1 mm deep. An electric field was applied along the *x*-axis by the WE and the CE placed at the right and left reservoirs, respectively. The glass micro-electrode, which was a reference electrode (RE), was translocated between the WE and CE in order to measure the potential difference relative to the WE. KCl and/or NaCl solutions of appropriate concentrations were used for the electric field measurements. Using a galvanostatic technique, the electrical potential in a solution was measured under constant-current conditions at room temperature. In order to measure the electrical potential at local points, we fabricated a glass capillary with a diameter of 1 μm by pulling a glass tube with an inner diameter of 0.8 mm using a glass puller (PC-100, NARISIGE Co., Tokyo, Japan). The diameter of the glass capillary tip determines the spatial resolution. In this capillary, a Ag-AgCl electrode, which was made from a Ag wire with a diameter of 0.3 mm (Niraco Corp., Tokyo, Japan), placed in an aqueous solution of sodium hypochlorite, was inserted in an agarose gel prepared with a 1.0 × 10^−3^ mol/L KCl solution (FUJIFILM Wako Pure Chemical Corp., Osaka, Japan). The agarose gel effectively acted to connect the electrode and the outer sample solution, suppressing leakage of the inner solution to the outer channel. Using a Ag-AgCl electrode packed in the glass capillary as a RE, the potential difference relative to the WE was measured under constant-current conditions between the WE and the CE using a galvanostat (VersaSTAT 4, Ametek Inc., Berwyn, PA, USA). The electrical potential distribution was locally measured by translocating the glass micro-electrode. In an equilibrated electrolyte solution, an electrode surface is known to be screened by highly concentrated electrolyte ions that form an EDL. In a KCl solution of 1 × 10^−3^ mol/L, the thickness of the EDL is equivalent to the Debye length of approximately 10 nm^3^, which is too small to measure using the present method. Herein, we measured the electric field in the bulk liquid in channels and evaluated the conductivity of the solution, avoiding observation of the EDL. In order to concentrate the electric field in the liquid, a narrow test section was produced in the middle of the channel, as shown in Fig. 1c. Under steady ionic current conditions assuming a uniform concentration, the electrical potential drop tends to concentrate in the narrower space. In such a narrow test section, which is far from the electrodes, the ionic current appears to obey Ohm’s law^14,16,22^. Thus, when an ionic current is dominated by electrophoretic transport, the conductance is proportional to the cross-sectional area *S* and inversely proportional to the channel length *L*. Therefore, the ionic current *I* is expressed in terms of the conductivity *σ* and applied electrical potential *V*, as follows: *I* = *σ*(*S*/*L*)*V*. In the present study, the above conjectures was tested by measuring the potential difference in electrolyte solutions.

### Solutions for electrical conductivity measurement

Next, the same measurements were performed on sample solutions with different concentrations. Using a 1.0 × 10^−3^ mol/L KCl for the inner solution in the glass capillary, the concentration of the outer solution was varied from 5.0 × 10^−4^ to 1.0 × 10^−2^ mol/L for KCl and NaCl. The conductivity was evaluated from the *I*-*V* characteristics measured locally in the test section. Using 1.0 × 10^−3^ mol/L KCl for the inner solution, the electrical conductivity of NaCl solutions was measured, where 1.0 × 10^−3^- and 1.0 × 10^−2^ mol/L NaCl solutions were prepared for the outer solution. Additionally, measurements were also performed using 1.0 × 10^−3^ mol/L NaCl as the inner solution and 1.0 × 10^−3^- and 1.0 × 10^−2^ mol/L KCl as the outer solution. The possibility of the glass micro-electrode measuring the electrical conductivity of the solutions is discussed. The potential difference between the WE and RE is caused by the concentration difference between the inner and outer solutions and the potential drop in the liquid. In the present method, the glass micro-electrode effectively senses potential differences in the sample solutions at the tip of the capillary. The glass tip and Ag-AgCl electrode inserted into the agarose gel enable us to measure the spatial distribution of potential difference. Otherwise, leakage of the inner solution into the channel would prevent stable measurements.

### Double-barreled glass micro-electrode for pH measurement

For further applications of the glass micro-electrode developed in the present study, as shown in Fig. 1d, the pH of sample solutions was determined using a double-barreled glass micro-electrode that exhibited asymmetric *I*-*V* characteristics. Based on the glass electrodes described above, a double-barreled capillary that was pulled by a glass tube puller was used for the sensor. Into the double-barreled capillary, both tips of which were filled with agarose gel prepared from a 1.0 × 10^−2^ mol/L KCl solution, a mixture of a buffer solution and a $1.0\times 1{0}^{-2}$ mol/L KCl solution was poured into one side and a 1.0 × 10^−2^ mol/L KCl solution was poured into the other side. The pH of the buffer solution was varied between 1.68 and 4.01 (Kishida Chemical Co., Ltd., Osaka, Japan). Ag-AgCl wires with a diameter of 0.3 mm were inserted into each solution. The Ag-AgCl wire in the mixed solution was used for the RE and CE, and the other solution was used for the WE. Oxalate buffer (pH 1.68), phthalate buffer (pH 4.01), phosphate buffer (pH 6.86), and borate buffer (pH 9.18) (Kishida Chemical Co., Ltd., Osaka, Japan) were used for the sample solutions. Under constant-current conditions of −1.0, 0.0, and 1.0 nA, the potential difference between the WE and RE was measured. The asymmetry of the solutions in the two capillaries was expected to produce asymmetry of the electrical conductivity, as if the glass capillary tip has proton selectivity, as shown in Fig. 1e. Depending on the direction of proton conduction, the electrical conductivity is different. When the Ag-AgCl electrode in the low-pH mixed solution acts as the anode, the protons in the inner solution are directed to the outer solution. On the other hand, when the polarity between the electrodes is reversed, proton conduction from the outer to the low pH inner solutions is suppressed due to highly concentrated protons in the glass capillary. As described above, an ionic current generated by the positively polarized WE is defined as positive. In this experiment, pulsed currents of ±1.0 nA were applied at an interval of several tens of seconds, which was the minimum required duration for steady-state measurement.

### Numerical analysis of electric fields in the channel

Electrical potential distributions were numerically evaluated by solving the Nernst-Planck and Poisson equations^18,50^. The electrostatic potential is related to the electrical charge density as follows:

1 $$\nabla \cdot {\varepsilon }_{0}\varepsilon {\bf{E}}=1000F\sum _{i}{z}_{i}{c}_{i},$$

where *ε*_0_ and *ε* are the dielectric constant of a vacuum and the relative constant of the solutions, respectively, **E** is the electric field, *z*_*i*_ and *c*_*i*_ are the valence and the molar concentration of the *i*th species, respectively, and *F* is the Faraday constant. **E** is represented by the electrostatic potential *ϕ* as **E** = −∇ *ϕ*. The ionic current density **J**_*i*_ is expressed as follows:

2 $${{\bf{J}}}_{i}=\frac{1000{F}^{2}{z}_{i}^{2}{D}_{i}{c}_{i}}{RT}{\bf{E}}-1000F{z}_{i}{D}_{i}{\rm{\nabla }}{c}_{i},$$

where *R* and *T* are the gas constant and the temperature, respectively. Herein, it is assumed that the sample solutions poured into the reservoirs and the test section do not undergo convection. The total current density is **J** = ∑_*i*_**J**_*i*_. In the steady state, the following is satisfied:

3 $$\nabla \cdot {\bf{J}}={\bf{0}}.$$

**Figure 7:**
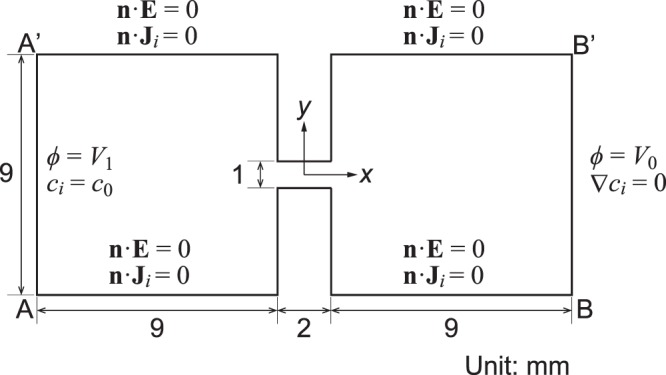
Dimensions and boundary conditions for two-dimensional FEM analysis to solve the Nernst-Planck and Poisson equations.

**Figure 8:**
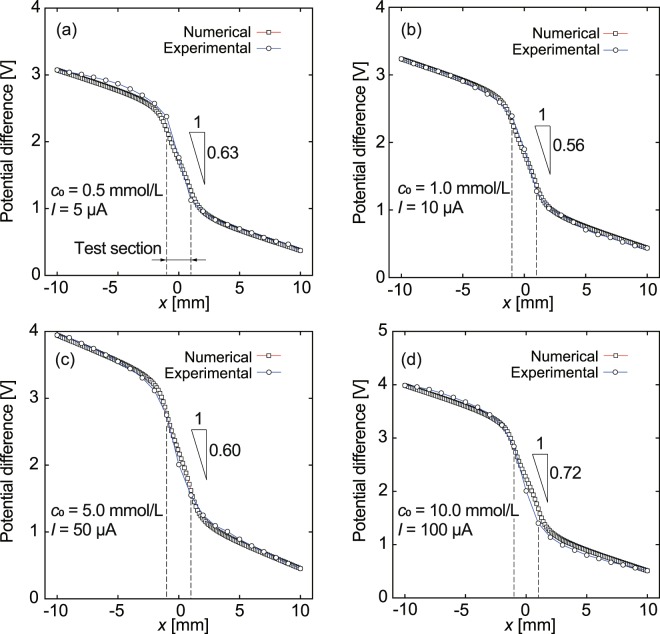
Experimental and numerical results for potential difference as a function of *x* along the channel: *c*_0_ = 5.0 × 10^−4^ (**a**), 1.0 × 10^−3^ (**b**), 5.0 × 10^−3^ (**c**), and 1.0 × 10^−2^ mol/L (**d**). The position of the test section is shown by the dashed lines.

The experimental system is replicated by a two-dimensional model, as shown in Fig. 7. The dimensions of the reservoirs were set to 9.0 mm in width and 9.0 mm in length, and the narrow test section is 1.0 mm wide and 2.0 mm long. Taking the experimental conditions into account, the concentrations of electrolyte ions were approximately uniform in the reservoirs and the test section, except near the electrically charged surfaces. For the boundary condition, the electrostatic potential was set to

4 $$\phi ={V}_{1}\,{\rm{o}}{\rm{n}}\,{{\rm{A}}{\rm{A}}}^{{\rm{{\prime} }}},$$

5 $$\phi ={V}_{0}\ \ \ {\rm{on}}\ \ {{\rm{BB}}}^{{\prime} },$$

and

6 $${\bf{n}}\cdot {\bf{E}}=0\ \ \ {\rm{on}}\ {\rm{AB}}\ {\rm{a}}{\rm{n}}{\rm{d}}\ {{\rm{A}}}^{{\prime} }{{\rm{B}}}^{{\prime} },$$

where **n** is the surface normal vector. The values of *V*_0_ and *V*_1_ were determined from the experimental results. Assuming a uniform distribution of ions in an equilibrium state without an external electric field, the molar concentration at the boundary AA′ was set to that of the bulk, *c*_0_, for each species, each of which was determined from the experimental conditions. On the other hand, ∇ *c*_*i*_ = 0 was set at the counter boundary BB′. On AB and A′B′, no-flux conditions, such that **n** ⋅ **J**_*i*_ = 0, were maintained. The parameters used in the numerical analysis are summarized in Table 3. Translocating the glass micro-electrode in the reservoirs and test section, the potential difference was locally measured. Figure 8 shows typical results for the potential difference as a function of the RE position in the experiment. The KCl concentrations were 5.0 × 10^−4^, 1.0 × 10^−3^, 5.0 × 10^−3^, and 1.0 × 10^−2^ mol/L. Numerical results obtained by the FEM analysis are also shown, which well reproduce the experimental results.

**Table 3:** Parameters used in numerical analysis.

*e* [C]	1.602 × 10^−19^
*F* [C/mol]	9.648 × 10^4^
*R* [J/mol K]	8.314
*T* [K]	298.15
${z}_{{{\rm{K}}}^{+}}$	+1
${z}_{{{\rm{Cl}}}^{-}}$	−1
${D}_{{{\rm{K}}}^{+}}$ [m^2^/s]	1.0 × 10^−9^
${D}_{{{\rm{Cl}}}^{-}}$ [m^2^/s]	1.0 × 10^−9^
*c*_0_ [mol/L]	5.0 × 10^−4^, 1.0 × 10^−3^, 5.0 × 10^−3^, and 1.0 × 10^−2^
